# Adherence to the MIND Diet and Cognition in Older Adults: A Post‐hoc Analysis of the MIND Trial

**DOI:** 10.1002/alz.090087

**Published:** 2025-01-09

**Authors:** Klodian Dhana, Puja Agarwal, Jennifer Ventrelle, Xiaoran Liu, Christy C Tangney, Vincent J Carey, Neelum T. Aggarwal, Konstantinos Arfanakis, Lisa L. Barnes

**Affiliations:** ^1^ Rush University Medical Center, Chicago, IL USA; ^2^ Rush Institute for Healthy Aging, Chicago, IL USA; ^3^ Rush Alzheimer’s Disease Center, Chicago, IL USA; ^4^ Rush University, Chicago, IL USA; ^5^ Harvard Medical School, Boston, MA, Boston, MA USA

## Abstract

**Background:**

The MIND randomized clinical trial demonstrated that the effect of the 3‐year MIND diet intervention on cognition was no different than the calorie‐restricted usual diet in overweight, non‐demented, older adults. However, compliance with dietary interventions may have differed among the participants within the intervention groups. We assessed the adherence to MIND diet (MIND score) and and evaluated the association with global cognition.

**Method:**

This study include 551 individuals with MIND diet score, covariates, and cognitive assessment at baseline and year 3. The MIND diet score (range 0‐14; a higher score reflects a healthier diet) was computed at baseline and during the follow‐up from responses to the 144‐item validated FFQ, and the average within individuals across the follow‐up was calculated. In addition, we computed the absolute (year 3 ‐ baseline) and relative change [((year 3 ‐ baseline)/baseline)*100]. Global cognition was derived from a standardized 12‐test cognitive battery. Regression models were adjusted by age, sex, race, APOE e4, education, cognitive activities, physical activity, BMI, total energy intake, and diet assignment group.

**Result:**

Higher adherence to the MIND diet across the study period was associated with significant improvements in global cognition scores. In the multivariable‐adjusted model, a 1‐point increase in the MIND diet score was associated with 0.027 (95%CI 0.004, 0.051) SD units higher in a 3‐year change in the global cognitive score. Compared to individuals with lower MIND diet scores (i.e., the first tertile), those in the third tertile had 0.140 (95%CI 0.033, 0.247) SD units higher in a 3‐year change in the global cognitive score. Similar associations were noted when we compared the relative changes of the MIND diet score with a 3‐year change in the global cognitive score. These associations (i.e., average MIND score, absolute, and relative changes) with cognition were not different between dietary interventions (*P* for interaction > 0.4).

**Conclusion:**

Adherence to the MIND diet varied across individuals in the MIND trial. A higher MIND diet score during 3 years of dietary interventions was associated with better cognitive performance in older adults.

